# Protective Effect of Epigallocatechin-3-gallate against Hepatic Oxidative Stress Induced by *tert*-Butyl Hhydroperoxide in Yellow-Feathered Broilers

**DOI:** 10.3390/antiox13101153

**Published:** 2024-09-24

**Authors:** Xinyan Ma, Junli Ni, Wei Wang, Yongwen Zhu, Yuqing Zhang, Mingfei Sun

**Affiliations:** 1 College of Veterinary Medicine, South China Agricultural University, Guangzhou 510642, China; dk051maxy@163.com (X.M.); wagnwei123@scau.edu.cn (W.W.); zhuyw0724@scau.edu.cn (Y.Z.); zhangyuqing0526@163.com (Y.Z.); 2 Institute of Animal Science, Guangdong Academy of Agricultural Sciences, Guangzhou 510640, China; 3 Institute of Animal Health, Guangdong Academy of Agricultural Sciences, Guangzhou 510640, China; 15988393006@163.com

**Keywords:** epigallocatechin-3-gallate, oxidative damage, antioxidant ability, Nrf2, PPARα, yellow-feathered broilers

## Abstract

Recent studies have shown that epigallocatechin-3-gallate (EGCG), as an effective antioxidant, could attenuate the oxidative damage, inflammation and necrosis in the liver in response to oxidative stress. The present study investigated whether oral administration of EGCG could effectively alleviate the hepatic histopathological changes and oxidative damage in yellow-feathered broilers induced by *tert*-butyl hydroperoxide (*t*-BHP). Broilers were exposed to 600 μmol *t*-BHP/kg body weight (BW) to induce oxidative stress by intraperitoneal injection every five days, followed by oral administration of different doses of EGCG (0, 20, 40 and 60 mg/kg BW) and 20 mg vitamin E (VE)/kg BW every day during 5–21 days of age. The results showed that *t*-BHP injection decreased (*p* < 0.05) body weight and the relative weight of the spleen; the enzyme activities of total antioxidant capacity (T-AOC), catalase (CAT) and total superoxide dismutase (SOD); and gene mRNA expressions of *nuclear factor erythroid 2-related factor 2* (*Nrf2*), *CAT*, *SOD1*, *SOD2* and *acetyl-CoA carboxylase (ACACA)*; as well as increased (*p* < 0.05) necrosis formation, malondialdehyde (MDA) content, reactive oxygen species (ROS)accumulation, and *peroxisome proliferator activates receptor-α* (*PPARα*) mRNA expression in the liver of yellow-feathered female broilers at 21 days of age. Treatment with 60 mg EGCG/kg BW orally could enhance antioxidant enzyme activities and reverse the hepatic damage induced by *t*-BHP injection by reducing the accumulation of ROS and MDA in the liver and activating the *Nrf2* and *PPARα* pathways related to the induction of antioxidant gene expression (*p* < 0.05). In conclusion, intraperitoneal injection of *t*-BHP impaired body growth and induced hepatic ROS accumulation, which destroyed the antioxidant system and led to oxidative damage in the liver of yellow-feathered broilers from 5 to 21 days of age. It is suggested that EGCG may play an antioxidant role through the *Nrf2* and *PPARα* signaling pathways to effectively protect against *t*-BHP-induced hepatic oxidative damage in broilers, and the appropriate dose was 60 mg EGCG/kg BW by oral administration.

## 1. Introduction

The output of broiler chickens in China reached 13.02 billion in 2023, including 9.43 billion white-feathered broiler chickens and 3.59 billion yellow-feathered broiler chickens [1]. With the improvement of modern breeding technology in poultry production, yellow-feathered broilers with the increased growth rate are becoming more susceptible to various stressors (e.g., intensive farming, nutritional imbalance and heat stress) [2,3]. The widespread challenge of stress, which induces oxidative damage, impairs growth performance and meat quality in broilers, leading to significant economic losses in commercial production [4]. *tert*-butyl hydroperoxide (*t*-BHP) has been widely used to generate excessive ROS, which lead to the occurrence of oxidative stress in animals [5,6]. Previous studies demonstrated that intraperitoneal injection of *t*-BHP triggers oxidative stress involving a series of complex cellular processes, such as ROS accumulation and lipid peroxidation in vitro and in vivo [6,7]. While the adverse effects of oxidative stress have been extensively studied in white-feathered broilers [8,9], there is limited research on its impact on yellow-feathered broilers, despite their different growth rates and stress responses. As with white-feathered broilers, yellow-feathered broilers as local broiler breeds in China have a lower growth rate but display a greater adaptive anti-stress capacity [10].

There are four major types of polyphenols in green tea: epigallocatechin-3-gallate (EGCG), epigallocatechin (EGC), epicatechin-3-gallate (ECG) and epicatechin (EC) [11]. Many studies have proven that EGCG, being the most active component, plays an important role in scavenging excessive ROS in a variety of tissues and cells [12,13]. The antioxidant efficacy of EGCG is exerted through direct mechanisms by phenolic hydroxyl groups scavenging reactive oxygen [14] and indirect mechanisms by inducing antioxidant enzymes through activating stress-related signaling pathways [13]. As indirect antioxidants, tea polyphenols can modulate the activities of redox-sensitive transcription factors, *Nrf2* and *PPAR* signal pathways, to suppress the stress-related oxidative damage in vitro [15,16]. However, a large body of evidence exists for the in vitro antioxidant effects of tea polyphenols, which are not always validated in vivo [16], suggesting that the efficacy of tea polyphenols in preventing oxidative stress remains controversial in vivo. Although increasing evidence has demonstrated that tea polyphenols have positive effects on growth and immunity in poultry [17,18], research has rarely focused on EGCG. It is necessary to explicate the protective effect of EGCG in broilers subjected to stressed challenge. *t*-BHP is commonly used as an oxidant to induce oxidative stress in chickens. Therefore, oxidative stress induced by *t*-BHP and the potential protective role of EGCG administration on oxidative damage in the liver of yellow-feathered broilers were investigated in this study.

## 2. Materials and Methods

### 2.1. Broilers, Management and Experimental Treatments

The animal protocols utilized in the current study were granted approval by the Institutional Animal Care and Use Committee of South China Agricultural University.

A total of 126 one-day-old yellow-feathered female broilers were acquired from a commercial hatchery (Wens Foodstuff Group Co., Ltd., Yunfu, China). The chicks at 5 days of age were randomly divided into 7 groups based on the similar BW of each group (18 broilers per group and 3 broilers per replicate, *n* = 6): control group (Control), intraperitoneal injection of 0.90% saline; *t*-BHP group (*t*-BHP), intraperitoneal injection of 600 μmol *t*-BHP/kg BW; EGCG groups, intraperitoneal injection of 600 μmol *t*-BHP/kg BW and given EGCG (purity 97%; Chengdu Herbpurify CO., LTD., Chengdu, China) at the doses of 20 (*t*-BHP + EGCG20), 40 (*t*-BHP + EGCG40) and 60 (*t*-BHP + EGCG60) mg/kg BW orally, respectively. Additionally, chickens were injected with 600 μmol *t*-BHP/kg BW and given 20 mg vitamin E/kg BW orally as a positive drug control group (*t*-BHP + VE). The time and dose of the intraperitoneal injection of *t*-BHP were conducted once every five days from d 5 to d 21 according to the previous study [19].

The care and management of the broilers were in accordance with the guidelines approved by the yellow-feathered broiler breed farm (Wens Foodstuff Group Co., Ltd., Yunfu, China). The broilers were housed in an electrically heated, thermostatically controlled room with windows, where the dimensions of the cages were 70 cm long, 70 cm wide and 40 cm high. The natural daylight was replaced by 18 h lighting provided by incandescent bulbs. The room temperature was initially maintained at 32 to 34 °C for the first three days and then gradually decreased by 2 to 3 °C per week until reaching a final temperature of 26 °C. The relative humidity of the room was maintained at 55–60%. Feed and water were available ad libitum during the experimental period. The diet (crumble food mixture) was formulated to meet the nutrient requirements for yellow-feathered broilers during 1–21 days of age according to the recommendations of NY/T 3645-2020. The ingredients and nutrient compositions in the diets are presented in Table 1.

### 2.2. Sample Collection and Processing

At 21 d of age, the BW of the broilers were weighed. Two broilers were selected based on the average BW in each cage to collect samples, which were pooled together based on replicate cage. They were bled by the wing vein with stainless-steel needles for whole blood. The serum and plasma were obtained by centrifuging the blood at 3500 rpm for 15 min at a temperature of 4 °C, followed by storage at −80 °C for subsequent analysis. Then, the broilers were subsequently killed. The liver, spleen and bursa of Fabricius were isolated from the broiler and subsequently weighed. The relative organ index was determined by calculating the ratio of the organ weight (g) to body weight (g). A liver sample from one broiler was fixed in 4% formaldehyde for histological analysis, while the rest of the liver samples were collected and frozen in liquid nitrogen and then stored at −80 °C for the activity of antioxidative enzyme and relative expression of gene mRNA analyses.

### 2.3. Serum Biochemical Parameters

The aspartate aminotransferase (AST) activity and albumin (ALB) content in serum were measured using a fully automatic biochemical analyzer (Mindray BS-380, Shenzhen, China).

### 2.4. Antioxidative Parameters in Plasma and Liver

Samples of the liver were homogenized using physiological saline solution (1:10, *v*/*v*) pre-cooled with ice. Subsequently, the homogenates were clarified by centrifugation at 3500 rpm for 10 min at a temperature of 4 °C. The activities of total antioxidant capacity (T-AOC, A015-3-1), total superoxide dismutase (T-SOD, A001-1) and catalase (CAT, A007-1-1) were quantified in plasma or liver samples using commercially available colorimetric kits provided by Nanjing Jiancheng Institute of Bioengineering. The content of malondialdehyde (MDA, A003-1) was also measured. The oxidative parameters in the liver were quantified as mg of protein, and the total protein content was determined using a commercial kit with bicinchoninic acid (W041-1-1, Nanjing Jiancheng Institute of Bioengineering, Nanjing, China).

### 2.5. Histopathological Analysis

After a fixation period of 24 h, the liver samples were dehydrated, embedded in paraffin, sectioned and stained with a standard hematoxylin–eosin (H&E) solution. The hepatic histopathology was observed and documented using a microscope (Eclipse E100 and DS-U3, Nikon, Tokyo, Japan).

### 2.6. Detection of Reactive Oxygen Species (ROS)

Immunocytochemistry was employed to determine the intracellular concentration of reactive oxygen species (ROS) in the liver samples utilizing an ROS measurement kit (Nanjing Jiancheng Bioengineering Institute, Nanjing, China) and visualized using a fluorescence microscope (Nikon, Tokyo, Japan). The pictures were obtained by the following steps: self-fluorescence of quenched liver tissues, dyeing, DAPI restaining of nuclei, sealing and image acquisition. The fluorescence was monitored using excitation at 488 nm and emission at 525 nm, while ROS generation was quantified based on the mean fluorescence intensity relative to that of the control.

### 2.7. Real-Time PCR Analysis

The Trizol Reagent was employed for the extraction of total RNA from freshly obtained liver samples [20]. The RNA concentration was determined by quantifying the absorbance of UV light at 260 nm using a spectrophotometer (NanoPhotometer-N60; IMPLEN, München, Germany), and the integrity of total RNA was checked through denatured RNA electrophoresis. Subsequently, 1 μg of total RNA was transcribed into cDNA using the Color Reverse Transcription Kit following the manufacturer’s protocol (EZBioscience, A0010CGQ, Beijing, China). qRT-PCR analysis was conducted on the ABI7500 system employing the PrimeScript™ RT Master Mix (Takara, RR036A, Beijing, China). *β-actin* expression served as an internal reference, and the geometric mean was utilized for normalizing the expression of the targeted genes [21]. The 2^−ΔΔCT^ method was used to determine the relative RNA expression levels. Detailed primer information can be found in Table 2.

### 2.8. Statistical Analysis

The data were subjected to one-way analysis of variance using SPSS 21.0 software. Duncan’s multiple range tests were employed for pairwise mean comparisons. All data are shown as means ± standard deviation. One replicate was considered as an experimental unit (*n* = 6). *p* < 0.05 was considered to be statistically significant. When treatment effects were significant (*p* < 0.05), differences among the means were tested by the LSD method.

## 3. Results

### 3.1. Body Weight and Relative Weight of Organs in Broilers

As shown in Table 3, the body weight (BW) and relative weight of the spleen of broilers treated with *t*-BHP injection were significantly decreased (*p* < 0.05) compared with those of broilers from the control group at d 21, while the oral treatment with EGCG increased (*p* < 0.05) linearly BW and relative weight of the spleen of *t*-BHP-induced broilers as the dose of EGCG rose from 20 to 60 mg/kg BW. Especially, the *t*-BHP + EGCG60 group effectively attenuated (*p* < 0.05) *t*-BHP-induced adverse effects on BW and relative weight of the spleen and restored them to the normal levels of the control and *t*-BHP + VE groups. There was no protective effect on the relative weight of the liver and bursa of Fabricius (*p* > 0.05).

### 3.2. Hepatic Histomorphology

**Figure 1:**
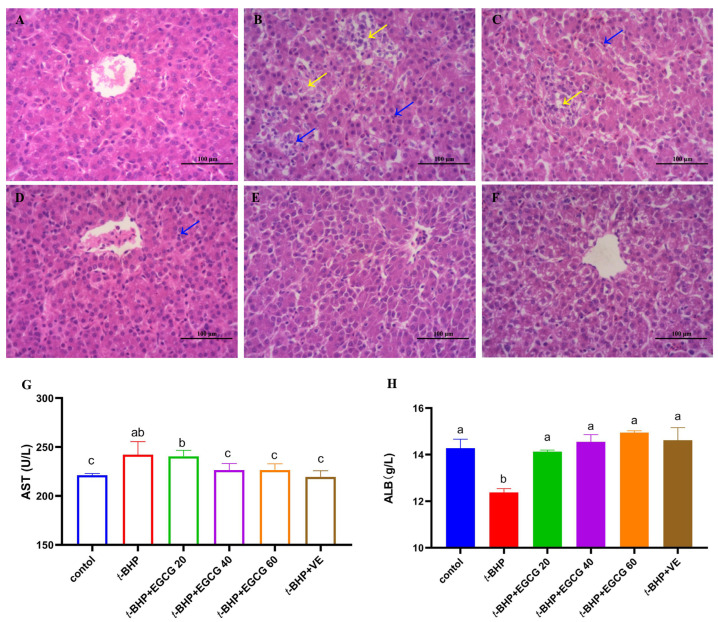
Effect on hepatic histomorphology of broilers damaged by *t*-BHP. Note: (**A**–**F**) indicate hepatic histomorphology (H&E) of broilers (200×). (**A**) represents the control group; (**B**) represents the experimental model of oxidative damage induced by *t*-BHP; (**C**–**F**) indicate treatment groups. Blue arrows indicate inflammation; yellow arrows signify necrotic areas. AST = alanine aminotransferase, ALB = albumin. (**A**) = control group, (**B**) = tBHP group, (**C**) = *t*-BHP + EGCG20, (**D**) = *t*-BHP + EGCG40, (**E**) = *t*-BHP + EGCG60, (**F**) = *t*-BHP + VE, (**G**) = AST activity in serum, (**H**) = ALB content in serum. The bar chart displays different letters to indicate statistically significant differences (*p* < 0.05), while the absence of letters or the presence of the same letters indicates a statistically insignificant difference (*p* > 0.05).

Prolonged injection of *t*-BHP resulted in the development of a necrotic area in the liver, accompanied by a significantly increased infiltration of inflammatory cells surrounding the centrilobular veins (Figure 1B). Treatment with EGCG mitigated the severity of the necrosis induced by *t*-BHP injection (Figure 1C–E). Additionally, *t*-BHP injection increased serum AST activity (Figure 1G) and decreased serum ALB content (Figure 1H) in broilers compared to the control and *t*-BHP + VE groups (*p* < 0.05), and these negative effects were eliminated by oral administration of 40–60 mg EGCG/kg BW in the *t*-BHP-treated broilers (*p* < 0.05).

### 3.3. ROS Accumulation in the Liver

**Figure 2:**
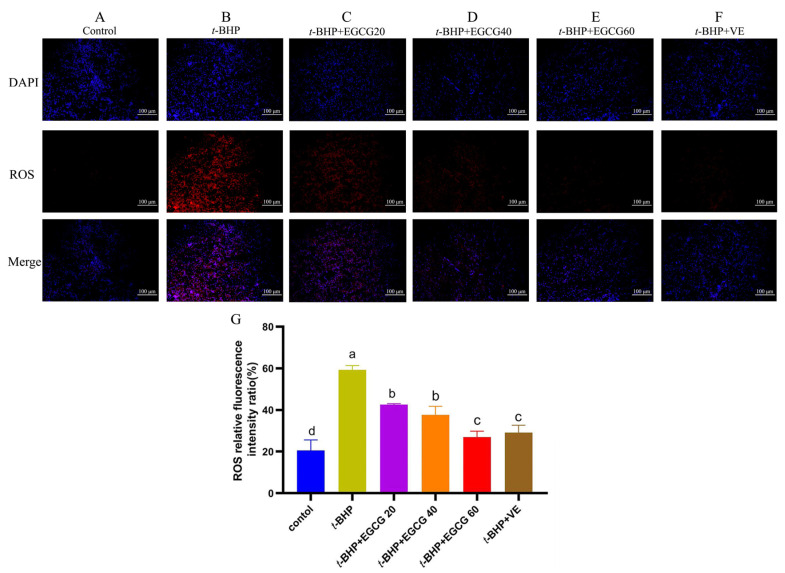
Effects of EGCG on ROS levels in liver tissue of broilers under oxidative stress. Note: (**A**–**F**) = the fluorescence intensity of ROS detected by immunofluorescence method in liver tissue (200×). (**G**) = the relative fluorescence intensity value of each group analyzed by ImageJ accessed in December 2023, which represents ROS levels. The bar chart displays different letters to indicate statistically significant differences (*p* < 0.05), while the absence of letters or the presence of the same letters indicates a statistically insignificant difference (*p* > 0.05).

As shown in Figure 2, *t*-BHP induced a remarkable increase (*p* < 0.05) of ROS formation in comparison with the control group, while treatment with EGCG resulted in decreased ROS accumulation in the liver compared to the *t*-BHP group (*p* < 0.05). There was no significant difference between the *t*-BHP + EGCG60 group and *t*-BHP + VE group in reducing liver ROS levels (*p* > 0.05), but the effect was better than the other levels of EGCG (*p* < 0.05).

### 3.4. Redox Status in Tissue

Compared with the control group, there was a significant decrease (*p* < 0.05) in the activity of T-AOC in plasma, and there were significant decreases in the activities of CAT and SOD and a significant increase (*p* < 0.05) in the MDA content in both plasma and the liver after *t*-BHP injection (Table 4). Given 60 mg EGCG/kg BW orally could enhance antioxidant enzyme activities and effectively prevent the antioxidant imbalance and lipid peroxidation in the liver induced by *t*-BHP (*p* < 0.05).

### 3.5. Gene mRNA Expression Related to Nrf2 and PPARα Pathways

**Figure 3:**
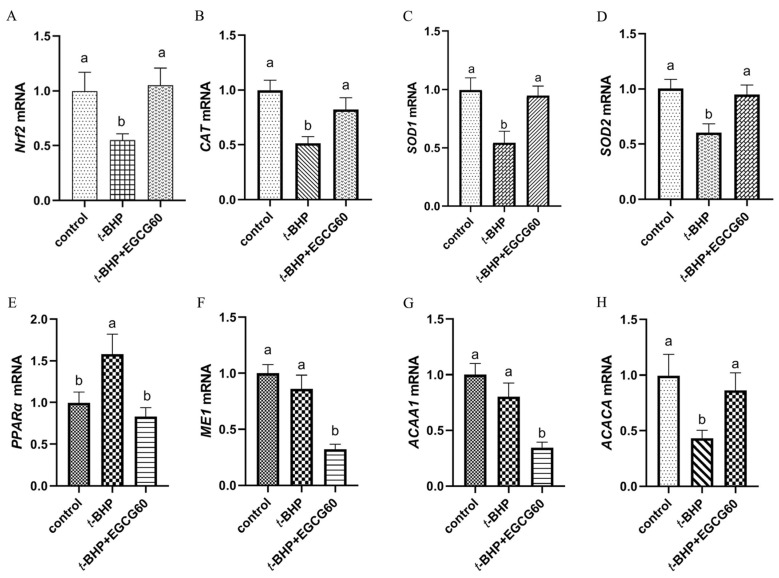
Effects of EGCG on gene expression related to antioxidant signaling pathway. Note: (**A**) Nrf2 = nuclear factor erythroid 2-related factor 2, (**B**) CAT = catalase, (**C**) SOD1 = superoxide dismutase 1, (**D**) = superoxide dismutase 2, (**E**) PPARα = peroxisome proliferator activates receptor-α, (**F**) ME1 = malic enzyme, (**G**) ACAA1 = acetyl-CoA acyltransferase1, (**H**) ACACA = acetyl-CoA carboxylase. The bar chart displays different letters to indicate statistically significant differences (*p* < 0.05). Conversely, the same letters on the bar chart represent statistically insignificant differences (*p* > 0.05).

Compared to the control group, *t*-BHP intraperitoneal injection decreased (*p* < 0.05) gene mRNA expressions of *Nrf2*, *CAT*, *SOD1*, *SOD2* and *ACACA* as well as increased (*p* < 0.05) peroxisome proliferator activates receptor-α (*PPARα*) mRNA expression in the liver, while administration of 60 mg EGCG/kg BW in broilers with *t*-BHP injection restored these gene expressions to the normal levels that showed no significant difference between the control group (Figure 3). Additionally, the treatment with *t*-BHP + EGCG up-regulated the expression levels of *Nrf2* and *ACACA* and down-regulated the expression levels of *PPARα*, *ME1* and *ACAA1* in the liver compared to the *t*-BHP group (*p* < 0.05). 

## 4. Discussion

With the improvement of breeding technology, modern broiler breeds with the rapid growth and high meat production are associated with increased metabolic activity and the production ofROS as byproducts during the grower period, which make broilers more susceptible to oxidative stress [22]. Additionally, environmental factors, such as high temperatures, poor ventilation, and high stocking densities, can induce the broiler’s antioxidant imbalance and oxidative stress [2,3]. The previous studies have demonstrated that *t*-BHP, a short-chain analog of a lipid hydroperoxide, has been utilized as an inducer of oxidative stress in vivo and in vitro [6,7]. In the present study, the model of oxidative stress was induced by intraperitoneal injection of 600 μmol *t*-BHP/kg BW as reported previously [7]. Compared to the broilers given saline, the body weight of yellow-feathered broilers given *t*-BHP decreased sharply, which was in line with the results in white-feathered broilers [23,24,25] under stressed stimulations. For one reason, *t*-BHP injection leads to the production of ROS, which can cause oxidative damage to cells, proteins, lipids and DNA, and then impairs overall health and growth. For the other reason, oxidative stress and inflammation from *t*-BHP might induce metabolic abnormalities of target tissues, leading to decreased nutrient metabolism and utilization to obtain sufficient nutrients for optimal growth and development of broilers. Our HE staining results of the histomorphology showed that vacuolation, inflammation and necrosis were increased in the hepatocytes of broilers after *t*-BHP injection, as characterized by the increased AST level and decreased ALB level in serum, suggesting that hepatic damage was triggered in broilers exposed to *t*-BHP. Our results showed that intraperitoneal injections of *t*-BHP significantly decreased the relative spleen weight of broilers but had no effect on the relative weight of the liver and bursa of Fabricius. The results are partly consistent with the previous research in broilers, which showed that oxidative stress-induced oxidative damage significantly suppressed the spleen development [26]. Furthermore, the exposure of *t*-BHP increased ROS production in the liver and pronounced lipid peroxidation, as evidenced by the increased MDA contents in serum and the liver, which are in line with the previous study in chick embryos [7] and rats [24]. In addition, the antioxidant ability of yellow-feathered broilers was reduced by *t*-BHP challenge, as evidenced by the decreased activities of SOD and CAT in plasma and the liver and the decreased T-AOC activity in plasma. These results are consistent with the previous findings that H_2_O_2_-induced oxidative stress generated excessive ROS and destroyed the antioxidant system in white-feathered broilers [24,27]. To sum up, it is noteworthy that *t*-BHP stimulation weakened the scavenging ability of ROS and induced oxidative damage in the liver of yellow-feathered broilers.

Many studies have highlighted the antioxidant role of tea polyphenols in reducing oxidative damage, and EGCG appears to exhibit the highest biological activity in polyphenols at the cellular level [13]. In the current study, the increasing doses of EGCG from 0 to 60 orally increased body weight and antioxidant ability by enhancing the activities of SOD and CAT in plasma and the liver of broilers under *t*-BHP-stressed conditions. It is supposed that EGCG promotes the activity of endogenous antioxidant enzymes, which helps to mitigate oxidative stress in liver tissues. Our results also confirm that EGCG exhibits protective effects against *t*-BHP-induced hepatic oxidative damage by reducing the accumulation of ROS and MDA in the liver. Previous studies have demonstrated that EGCG may inhibit enzymes like NADPH oxidase [28,29], which are involved in the production of ROS, thereby reducing their overall levels in tissues and cells. As mentioned before, the antioxidant effects of EGCG display a dose-dependent manner in relation to scavenging the generation of ROS [30]. Furthermore, there is evidence suggesting that the effect of EGCG on the reduction in ROS production in the liver is dose-dependent, while the maximum protective effect of EGCG was noted at a dose of 60 mg/kg BW, associated with the reduction in hepatic ROS production and structural damage. As reported in the activity of key antioxidant enzymes, EGCG can enhance SOD and CAT activities and pronounce antioxidant effects following the increased injected doses. Other studies on the protection of EGCG against oxidative stress induced by carbon tetrachloride [31] and lead [32] have also reported similar data. It is noteworthy that the higher dose of 60 mg EGCG/kg BW orally can effectively prevent a ROS increase and restore the *t*-BHP-induced decreases in structural integrity and antioxidant enzyme activities to the normal levels of broilers given VE orally. It is confirmed that, at higher concentrations, EGCG can significantly reduce ROS levels, offering substantial protection against oxidative damage in hepatocytes [33,34]. However, some reports indicated that the roles of polyphenols and their metabolites related to the antioxidant effect were not always validated in vivo [35]. For example, the administration of excessively high doses of EGCG may potentially result in cellular damage and adverse effects under specific circumstances, potentially leading to increased oxidative stress if not balanced by antioxidant mechanisms [36].

EGCG can influence various signaling pathways that regulate oxidative stress responses, including the *Nrf2* and *PPARα* pathways, which help in the cellular defense mechanisms against ROS. The transcription factor *Nrf2*, widely recognized for its pivotal role in the antioxidant system, plays a crucial function in the cellular stress response by binding to antioxidant response elements [37]. Previous studies have reported that oxidative stress induced by an increase in ROS production was associated with the inhibition of the *Nrf2* pathway in muscle of white-feathered broilers [38]. In the present study, the *Nrf2* mRNA expression was significantly decreased in broilers treated with *t*-BHP, while intragastric administration of 60 mg EGCG/kg BW could up-regulate *Nrf2* mRNA expression and target antioxidant gene mRNA expression (*CAT*, *SOD1* and *SOD2*) in the liver of broilers subjected to *t*-BHP-induced oxidative stress, which contributed to strengthening the antioxidant defense system of broilers. These positive results from EGCG were in agreement with both HepG2 cells and mice liver [39]. The previous study reported that black tea polyphenols can enhance the expression of antioxidant enzymes in the liver of mice via activation of the *Nrf2-ARE* pathway [15]. In addition, more studies have paid particular attention on regulating *PPARα* expression and activity involved in controlling oxidative stress and inflammation [40]. Our study showed that *t*-BHP-induced oxidative stress up-regulated *PPARα* mRNA expression and down-regulated *ACACA* mRNA expression in the liver of broilers, which recovered to normal levels by oral administration of EGCG. Moreover, EGCG intervention could reduce the *ME1* and *ACAA1* mRNA expression related to the oxidation of fatty acids in the liver of broilers under *t*-BHP stimulation, which also could contribute to the decreased lipid peroxidation in broilers given EGCG. Thereby, these data implied that EGCG could prevent hepatic oxidative damage from ROS accumulation by regulating the *Nrf2* and *PPARα* pathways related to the induction of antioxidant gene expression, but the exact mechanism of the crosstalk between *Nrf2* and *PPARα* needs further investigation.

## 5. Conclusions

In conclusion, intraperitoneal injection of *t*-BHP impaired body growth, induced hepatic ROS accumulation and oxidative damage and destroyed the antioxidant system in the liver of yellow-feathered broilers from 5 to 21 days of age. The oral administration of 60 mg EGCG/kg BW exhibited protective effects against *t*-BHP-induced hepatic oxidative damage by reducing the accumulation of ROS and MDA in the liver, which involved the *Nrf2* and *PPARα* pathways related to the induction of antioxidant gene expression.

## Figures and Tables

**Table 1:** Main ingredients and nutrient analysis of the diets.

Ingredients	Content (%)
Corn	60.76
Soybean meal (46% crude protein content)	28.32
Corn gluten meal (61% crude protein content)	5.15
Soybean oil	1.00
Lysine-HCl	0.30
DL-Methionine	0.26
Monocalcium phosphate	2.10
Limestone	0.90
Salt	0.21
Vitamins and minerals ^1^	1.00
Calculated values (%) ^2^	
Crude protein	21.5
Crude fat	3.40
Crude fiber	3.39
Calcium	1.00
Non-phytic acid phosphor	0.46
Metabolizable energy (kcal/kg of diet)	3003

Note: ^1^ The provided amount of vitamins per kilogram of diet includes 15,000 IU of vitamin A, 300 IU of vitamin D3, 20 IU of vitamin E, 36 mg of vitamin K, 1.8 mg of vitamin B1, 9 mg of vitamin B2, 3.5 mg of vitamin B6, 0.01 mg of vitamin B12, 60 mg niacin (vitamin B3), 0.15 mg biotin (vitamin H), 16 mg D-pantothenic acid (vitamin B5), 0.55 mg folic acid (vitamin B9), 500 mg choline, Mn (manganese) 80 mg, Zn (zinc) 65 mg, Fe (iron) 80 mg, Cu (copper) 8 mg, Se (selenium) 0.15 mg and I (iodine) 0.35 mg. ^2^ Nutrient levels were calculated values.

**Table 2:** The information of the used primers.

Genes	Accession Number	Primer Sequences (5′→3′)	Product Size (bp)
*β-actin*	NM_205518	F: TGCGTGACATCAAGGAGAAG R: TGCCAGGGTACATTGTGGTA	300
*CAT*	NM_001031215.2	F: AGCAGGTGCCTTTGGCTATT R: TCCAGCAACAGTGGAGAACC	121
*SOD1*	NM_205064.1 125	F: CCGGCTTGTCTGATGGAGAT R: TGCATCTTTTGGTCCACCGT	125
*SOD2*	NM_204211.2	F: CGCTGGCAAAAGGTGATGTT R: GCGAAGGAACCAAAGTCACG	172
*Nrf2*	XM_046921130.1	F: CATAGAGCAAGTTTGGGAAGAG R: GTTTCAGGGCTCGTGATTGT	105
*PPARα*	NM_001001464	F: CAAACCAACCATCCTGACGAT R: GGAGGTCAGCCATTTTTTGGA	64
*ACACA*	AB160952.1	F: CTCGTTCGGTTAGGGCAGAGG R: CCCGTCCCAGCACCTTGTT	177
*ACAA1*	NM_001197288.1	F: CCAGCATACTGACAGCCCAA R: TCCCACTTGCACATCAGACC	170
*ME1*	NM_204303.2	F: AGCATTACGGTTTAGCATTTCGG R: CAGGTAGGCACTCATAAGGTTTC	240

**Table 3:** Effects of EGCG on the body weight and relative weight of organs of chickens under oxidative stress.

Treatment	Body Weight (g)	Spleen Index (%)	Liver Index (%)	Bursa of Fabricius Index (%)
Control group	508 ± 21 ^a^	0.20 ± 0.01 ^a^	3.36 ± 0.10	0.30 ± 0.02
*t*-BHP group	442 ± 30 ^c^	0.16 ± 0.01 ^c^	3.32 ± 0.03	0.25 ± 0.01
*t*-BHP + EGCG20	456 ± 30 ^c^	0.16 ± 0.01 ^bc^	3.17 ± 0.06	0.27 ± 0.02
*t*-BHP + EGCG40	477 ± 26 ^bc^	0.16 ± 0.01 ^bc^	3.2 ± 0.08	0.29 ± 0.02
*t*-BHP + EGCG60	507 ± 33 ^a^	0.17 ± 0.01 ^b^	3.33 ± 0.09	0.32 ± 0.02
*t*-BHP + VE	498 ± 38 ^ab^	0.14 ± 0.01 ^bc^	3.16 ± 0.06	0.28 ± 0.01
*p* value	0.001	0.002	0.324	0.269

Note: Data are expressed as mean ± STD (*n* = 6); spleen index % = spleen weight/body weight × 100, liver index % = liver weight/body weight × 100, bursa of Fabricius index % = bursa of Fabricius index/body weight × 100. Different letters on the shoulder within the same column indicate statistically significant differences, *p* < 0.05; the same letters or no letters on the shoulder in the same column indicate a statistically insignificant difference, *p* > 0.05.

**Table 4:** Effects of EGCG on antioxidant indexes in plasma and liver of broilers under oxidative stress.

Treatments	Plasma				Liver		
	T-AOC, nmol/mL	T-SOD, U/mL	CAT, U/mL	MDA, nmol/mL	T-SOD, U/mgprot	CAT, U/mgprot	MDA, nmol/mgprot
Control	1.59 ± 0.15 ^a^	534 ± 24 ^a^	14.93 ± 1.50 ^a^	1.77 ± 0.32 ^b^	638 ± 78 ^a^	3.47 ± 0.69 ^a^	0.43 ± 0.05 ^b^
*t*-BHP group	1.27 ± 0.13 ^b^	451 ± 32 ^b^	11.76 ± 1.40 ^c^	2.53 ± 0.64 ^a^	509 ± 22 ^d^	1.44 ± 0.39 ^d^	0.66 ± 0.06 ^a^
*t*-BHP + EGCG20	1.56 ± 0.26 ^a^	488 ± 49 ^ab^	11.60 ± 1.71 ^c^	1.89 ± 0.23 ^b^	590 ± 19 ^bc^	1.74 ± 0.58 ^cd^	0.63 ± 0.10 ^a^
*t*-BHP + EGCG40	1.61 ± 0.16 ^a^	495 ± 34 ^ab^	12.32 ± 1.43 ^bc^	1.97 ± 0.29 ^b^	623 ± 27 ^ab^	2.35 ± 0.52 ^b^	0.59 ± 0.10 ^a^
*t*-BHP + EGCG60	1.56 ± 0.07 ^a^	536 ± 41 ^a^	14.53 ± 1.91 ^a^	1.67 ± 0.34 ^b^	635 ± 25 ^a^	2.73 ± 0.22 ^b^	0.48 ± 0.02 ^b^
*t*-BHP + VE	1.60 ± 0.09 ^a^	536 ± 85 ^a^	14.65 ± 2.08 ^a^	1.67 ± 0.17 ^b^	559 ± 13 ^c^	2.77 ± 0.38 ^b^	0.49 ± 0.04 ^b^
*p* value	0.009	0.040	0.002	0.003	0.001	0.002	0.031

Note: T-AOC = total antioxidant capacity, CAT = catalase, SOD = superoxide dismutase, MDA = malondialdehyde. Different letters on the shoulder within the same column indicate statistically significant differences, *p* < 0.05; The same letters on the shoulder in the same column indicate a statistically insignificant difference, *p* > 0.05.

## Data Availability

The data can be provided upon request.
